# Comprehensive quality evaluation of different types of Gardeniae Fructus (*Zhizi*) and *Shuizhizi* based on LC-MS/MS

**DOI:** 10.3389/fpls.2024.1346591

**Published:** 2024-02-27

**Authors:** Huimin Qian, Yan Hu, Zhiwei Wang, Aoyu Ren, Haiwen Zhang, Shanshan Chu, Huasheng Peng

**Affiliations:** ^1^ School of Pharmacy, Anhui University of Chinese Medicine, Hefei, China; ^2^ State Key Laboratory for Quality Ensurance and Sustainable Use of Dao-di Herbs, National Resource Center for Chinese Materia Medica, China Academy of Chinese Medical Sciences, Beijing, China; ^3^ Research Unit of DAO-DI Herbs, Chinese Academy of Medical Sciences (2019RU57), Beijing, China; ^4^ Department of Traditional Chinese Medicine, Anhui Province Key Laboratory of Research and Development of Chinese Medicine, Hefei, China

**Keywords:** Gardeniae Fructus (*Zhizi*), *Shuizhizi*, iridoids, quality evaluation, distribution difference

## Abstract

Gardeniae Fructus (*Zhizi*) serves as both a medicinal and edible substance and finds widespread use in various industries. There are often two kinds of medicinal materials in the market: *Zhizi* and *Shuizhizi*. Typically, *Zhizi* with small, round fruit is used for medicinal purposes, while *Shuizhizi*, characterized by large, elongated fruit, is employed for dyeing. Market surveys have revealed a diverse range of *Zhizi* types, and modern research indicates that *Shuizhizi* contains rich chemical components and pharmacological activities. In this study, we collected 25 batches of *Zhizi* and *Shuizhizi* samples, categorizing them based on appearance into obovate and round fruits, with seven length grades (A–G). Using the ultra-high performance liquid chromatography coupled with triple quadrupole mass spectrometry (UHPLC-QQQ-MS/MS) method, we simultaneously quantified 13 main chemical components in fruits of *Gardenia* species. In addition, we compared the weight percentage of the pericarp, flesh, and seeds parts of samples with different traits, and quantified 13 chemical components in different parts. Results indicated that, aside from a few instances of overlapping fruit size ranges, *Shuizhizi* generally exhibits larger and longer dimensions than *Zhizi*. The weight proportion of the *Shuizhizi* pericarp is often higher than that of the *Zhizi* pericarp. Quantitative results highlighted significant differences in the chemical component content between *Zhizi* and *Shuizhizi*, with *Shuizhizi* generally containing higher levels of iridoids. The PCA and OPLS-DA analysis distinctly divided *Shuizhizi* and *Zhizi*, among which three iridoids, two organic acids, and one flavonoid made significant contributions to their classification. Cluster heatmap analysis also demonstrated complete separation between *Zhizi* and *Shuizhizi*, with clear distinctions among *Zhizi* samples from different origins. The distribution of the 13 chemical components in different *Zhizi* and *Shuizhizi* parts remained consistent, with iridoids and pigments concentrated in the seeds and flesh, and two organic acids and one flavonoid enriched in the pericarp. In summary, this study contributes valuable insights for classifying *Zhizi* and offers guidance on the rational use of *Shuizhizi* and the different parts of *Zhizi*.

## 1 Introduction

Gardeniae Fructus known as *Zhizi* in Chinese, serves as a common Chinese herbal medicine and finds applications in pharmaceuticals, food, cosmetics, and dye industries (Chen et al., 2020b; Tian et al., 2022; Jin et al., 2023). Its historical use dates back over 2000 years to the Han Dynasty, when it was documented as both a medicinal and dyeing agent. Traditionally, small, round fruits are designated for medicinal purposes, referred to as *Zhizi* or *Shanzhizi* in Chinese. Conversely, large, elongated fruits known as “*Fu shi Zhizi*” or *Shuizhizi* are used exclusively for dyeing (Liu and Peng, 2016). Currently, two distinct forms of medicinal materials persist in the market: *Zhizi*, characterized by smaller fruits, and the slightly larger *Shuizhizi*, aligning with historical herbal medicine records. *Zhizi* is derived from the dried ripe fruit of *Gardenia jasminoides* Ellis (Committee for the Pharmacopoeia of PR China, 2020), while *Shuizhizi* originates from various sources, primarily from *G. jasminoides* f. *longicarpa* Z. W. Xie & M. Okada or *G. jasminoides* var. *grandiflora* Nakai (Xie, 1991; Liu et al., 2023).

*Zhizi* and *Shuizhizi* are rich in active chemicals, including iridoids (e.g., geniposide, geniposidic acid, shanzhiside, deacetylasperulosidic acid methyl ester, genipin 1-gentiobioside, genipin), pigments (e.g., crocin I, crocin II), organic acids (e.g., caffeic acid, chlorogenic acid, neochlorogenic acid, protocatechuic acid), and flavonoids (e.g., isoquercitrin, rutin) (Xiao et al., 2017; Ye et al., 2022b; Zhang et al., 2024). The abundant chemical components in *Zhizi* exhibit various pharmacological effects such as hepatoprotection (Fan et al., 2020), anti-inflammatory (Zhou et al., 2019), anti-hyperglycemia (Zhou et al., 2023), anti-depression (Zou et al., 2021), and anti-allergy (Pyun et al., 2021) activities. *Zhizi* is commonly used in traditional Chinese medicine for clearing heart fire, relieving restlessness, eliminating damp heat, and cooling blood detoxification (Committee for the Pharmacopoeia of PR China, 2020). In contrast, *Shuizhizi* is primarily utilized for pigment extraction and is not employed in traditional Chinese medicine. Despite their historical distinctions, modern studies reveal similarities in the chemical components of *Zhizi* and *Shuizhizi*, albeit with slight variations in content (Liu et al., 2023). Recent pharmacological studies suggest hepatoprotective properties of *Shuizhizi* (Fu et al., 2001). At the same time, studies have suggested that *Shuizhizi* shared anti-hypertensive effects similar to *Zhizi* (Chen et al., 2017; Hou et al., 2021), sparking curiosity about its potential medicinal significance.

In recent years, advanced analytical methods such as ultra-flow liquid chromatography coupled with electrospray ionization triple quadrupole mass spectrometry (UFLC-Q-TRAP-MS/MS) and ultra-performance liquid chromatography (UPLC) have been employed for quantitative analysis and fingerprinting of *Zhizi*, providing insights into optimal harvesting times and quality evaluations from different regions (Shan et al., 2019; Cao et al., 2021a; Li et al., 2021). Additionally, researchers have utilized ultrahigh-performance liquid chromatography/quadrupole-orbitrap mass spectrometry (UHPLC/Q-Orbitrap MS) and UHPLC-triple quadrupole-linear ion trap mass spectrometry (UHPLC-QTRAP-MS) methods to identify thirteen differential markers and quantify metabolites in *Zhizi* and *Shuizhizi*, aiding in their differentiation (Zhang et al., 2024).

Recently, increasing attention has been given to the relationship between the appearance and quality of Chinese medicinal materials such as Peucedani Radix and Cimicifugae Rhizoma (Chu et al., 2020; Ma et al., 2023). Previous studies on *Zhizi* indicate a significant correlation between its appearance traits and its quality and medicinal properties. For example, as the color of *Zhizi* deepens into red, it indicates higher levels of crocin I. Conversely, a more yellow hue signifies had higher concentrations of total phenolic acids and rutin within the fruit (Fu et al., 2020a; Ye et al., 2022a). Another study revealed disparities in the chemical composition of *Zhizi* across various fruit ridges (Tang et al., 2020).

The existing commodity specification standard for Gardeniae Fructus primarily categorizes it into different grades, based on factors like fruit maturity, fullness, and color (Zhang et al., 2019). Whereas, due to long-term artificial cultivation, the shape, size and other characteristics of the *Zhizi* have undergone significant variation. Market transactions often rely on the shape and size of Gardeniae Fructus for grade evaluations, where smaller, round fruits are considered higher quality. However, the diversity in *Zhizi’s* characteristics and its impact on quality warrant further research. This paper aims to contribute to this understanding by collecting different types of *Zhizi* and *Shuizhizi*, utilizing UHPLC-QQQ-MS/MS for the simultaneous determination of 13 active components (six iridoids, four organic acids, two pigments, and one flavonoid) and analyzing those major active components in the whole fruit and different parts of *Zhizi* and *Shuizhizi*. The objective is to comprehensively evaluate the quality of *Zhizi* and *Shuizhizi* with different characteristics.

## 2 Materials and methods

### 2.1 Chemicals and reagents

The standard compounds (i.e., geniposide, genipin, shanzhiside, geniposidic acid, caffeic acid, chlorogenic acid, neochlorogenic acid, crocin I, crocin II, and isoquercitrin) were procured from Chengdu Push Biotechnology Co., Ltd. (Chengdu, China). Genipin 1-gentiobioside was obtained from Chengdu Desite Biotechnology Co., Ltd. (Chengdu, China), and deacetylasperulosidic acid methyl ester and protocatechuic acid were purchased from Shanghai Yuanye Biotechnology Co., Ltd. (Shanghai, China). The purity of each quantitatively analyzed compound exceeded 98%, with the exception of geniposidic acid (purity ≥ 95%). The chemical structures of all reference standards are presented in **Supplementary Figure 1**.

LC-MS-grade methanol and acetonitrile were sourced from Merck (Darmstadt, Germany), while formic acid (LC-MS grade) was acquired from Aladdin (California, United States). Ultrapure water was supplied by a Milli-Q system (Millipore, United States). All the other reagents used were of analytical grade.

### 2.2 Sample collection and classification

**Figure 1:**
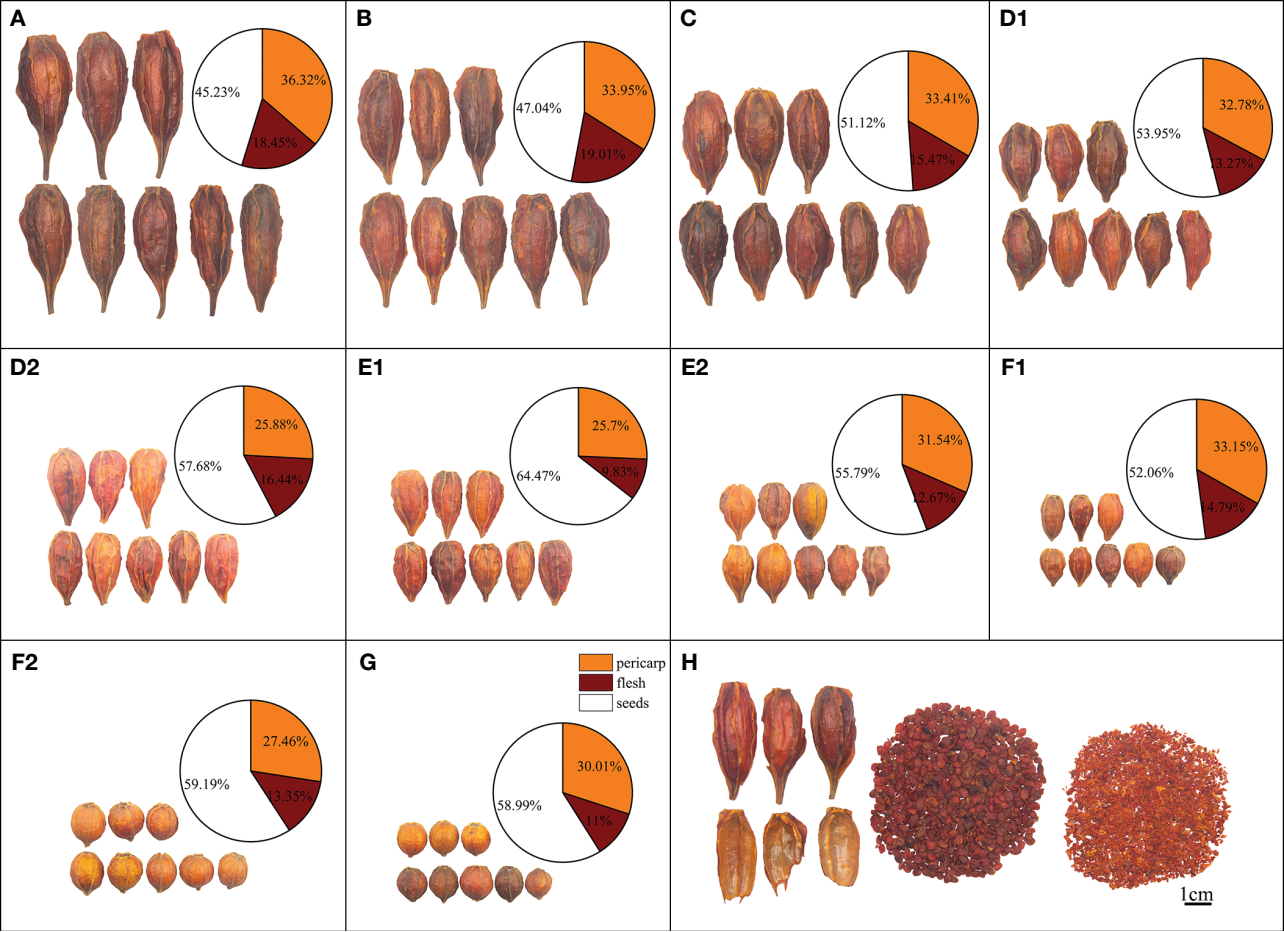
Fruits of *Gardenia* species (*Zhizi* and *Shuizhizi*) with different morphological traits and parts, as well as the weight ratio pie graphs of different parts of them. **(A)** Obovate Jiangxi *Shuizhizi* (5.3 cm < L ≤ 6.3 cm); **(B)** Obovate Jiangxi *Shuizhizi* (4.3 cm < L ≤ 5.3 cm); **(C)** Obovate Jiangxi *Shuizhizi* (3.3 cm < L ≤ 4.3 cm); **(D1)** Obovate Jiangxi *Shuizhizi* (2.7 cm < L ≤ 3.3 cm); (**D2)** Obovate Fujian *Zhizi* (2.7 cm < L ≤ 3.3 cm); (**E1)** Obovate Fujian *Zhizi* (2.1 cm < L ≤ 2.7 cm); (**E2)** Obovate Jiangxi *Zhizi* (2.1 cm < L ≤ 2.7 cm); **(F1)** Obovate Jiangxi *Zhizi* (1.5 cm < L ≤ 2.1 cm); **(F2)** Round Jiangxi *Zhizi* (1.5 cm < L ≤ 2.1 cm); **(G)** Round Jiangxi *Zhizi* (0.8 cm < L ≤ 1.5 cm); **(H)** Different parts of *Shuizhizi* fruit (left to right: pericarp, seeds, and flesh).

Twenty-five batches of *Gardenia* species samples, comprising nineteen batches of *Zhizi* samples and six batches of *Shuizhizi* samples, were collected from the Jiangxi and Fujian provinces. The detailed sample information is provided in **Supplementary Table 1**. Prof. Huasheng Peng of the National Resource Center for Chinese Materia Medica, China Academy of Chinese Medical Sciences, authenticated all samples. The samples were categorized based on obovate and round morphological characteristics. Obovate and round fruits were distinguished by the largest fruit diameter’s location — upper and middle parts of the whole fruit, respectively. Additionally, all samples were further classified into seven length grades (A–G) based on fruit length, measured from the fruit base (excluding, the fruit stalk) to the top narrowing place. **Figure 1** illustrates the samples with different morphological features.

### 2.3 Fruit morphological measurements

#### 2.3.1 Measurement of fruit length, maximum diameter, weight, and volume

Fifteen fruits were randomly selected per batch. Using a vernier caliper, the length between the base (excluding the stem) and the narrow apex of each fruit, along with the maximum diameter, were measured. Both length and maximum diameter measurements were repeated three times for each fruit. Subsequently, the ratio of the length to the maximum diameter was then calculated for each fruit.

For each batch of the *Zhizi* samples, random division into three groups, each containing an equal number of seeds, was performed. An electronic balance was used to measure the weight of each group. After filling a beaker with fine sand (120 mesh), the fruits were buried in the sand. The spilled fine sand’s volume was determined using a volumetric cylinder, representing the volume of each fruit group. Weight and volume measurements were repeated three times, and the density of each fruit was calculated as the weight-to-volume ratio.

#### 2.3.2 Determination of the weight ratio of pericarp, flesh, and seeds in fruit

Nine fruits, originating from various geographical regions and possessing different length grades, were randomly selected. These fruits were manually divided into three parts: the pericarp, flesh, and seeds, with three parallels for each part. The weight of each part was measured, and the weight ratio of different parts in relation to the whole fruit was calculated.

### 2.4 Quantitative analysis of chemical compounds

#### 2.4.1 Standard and sample preparation

The standard compounds were accurately weighed and individually dissolved in 70% methanol to prepare stock solutions of approximately 1.0 mg/mL, except for crocin I and crocin II, which were stored in 0.5 mg/mL stock solutions. These stock solutions were then diluted with 70% methanol to construct calibration curves. The resulting standard solution was stored at –20°C until injection.

The dried sample powder (60 mesh), precisely weighed at 0.1 g, was placed in conical flasks and combined with 25 mL of 70% methanol. After weighing, the mixture underwent ultrasonication at 25°C (100 W, 40 kHz) for 30 min. Subsequently, the solutions were cooled to room temperature, and the same solvent was added to each solution to compensate for the weight loss. The supernatant was collected after centrifugation at 5000 r/min for 10 min. Finally, the solution was filtered through a 0.22 µm Millipore filter and stored at 4°C before injection.

#### 2.4.2 LC-MS/MS equipment and conditions

Chromatographic analysis was conducted on an LC-30A ultra-high-performance liquid chromatograph (Shimadzu, Japan), equipped with an Acquity UPLC BEH C18 column (100 mm × 2.1 mm, 1.7 µm) and a BEH C18 VanGuard pre-column (2.1 mm × 5 mm, 1.7 µm). The mobile phase consisted of 0.2% formic acid in H_2_O (A) and acetonitrile (B). The gradient elution procedure was as follows: 0–2 min, 4%–6% B; 2–5 min, 6%–8.5% B; 5–6 min, 8.5%–12% B; 6–8 min, 12%–15% B; 8–14 min, 15%–18% B; 14–16 min, 18%–24% B; 16–18 min, 24%–100% B; 18–19 min, 100%–4% B; and 19–22 min, 4% B. The mobile phase operated at a flow rate of 0.25 mL/min, and the injection volume was 2 μL. The column temperature was maintained at 40°C.

All analytes were detected by a Q-TRAP™ 4500 MS/MS system (AB Sciex, United States) equipped with an electrospray ionization (ESI) source for mass spectrometry detection. Compound-dependent MS parameters, including precursor ion (Q1), production (Q3), declustering potential (DP), and collision energy (CE) were designed and optimized using the multiple reaction monitoring (MRM) modes. Other MS parameters were set as follows: source temperature, 500°C; ion source gas 1, 40 psi; ion source gas 2, 40 psi; curtain gas, 40 psi; and dwell time, 50 ms. Data was acquired and processed using AB Sciex Analyst 1.5.2 software (AB Sciex, United States).

### 2.5 Method validation

A series of standard solutions of appropriate concentrations were prepared to construct a calibration curve. The limits of detection (LODs) and quantification (LOQs) were defined using standard solutions as signal-to-noise (S/N) concentrations of 3 and 10, respectively. Testing of the 13 analytes within Sample S5 was repeated six times per day to evaluate the intra-day precision. To assess inter-day precision, the same solution was analyzed for three consecutive days. The repeatability of the method was verified by testing sample solution S5 six times. Aliquots from the same solution were injected at 0, 2, 4, 8, 12, and 24 h, respectively, and the relative standard deviation (RSD) values were calculated to validate stability. In the recovery test, standard analytes were added to six samples taken from S5 at amounts equivalent to those of the samples and then processed and analyzed following the procedure described as described above.

### 2.6 Data analysis

All data were expressed as mean ± standard deviation (Mean ± SD). Statistical evaluation was conducted using a one-way analysis of variance (ANOVA) and the Duncan’s multiple range test with IBM SPSS 26.0 Statistics software (SPSS, Inc., United States). Statistically significant differences were considered for a p-value lower than 0.05. Significantly different data (p < 0.05) were denoted by different letters. The histogram and pie graphs were created using Origin 2021 software (OriginLab, United States). Principal component analysis (PCA) scores and orthogonal partial least squares discriminant analysis (OPLS-DA) were performed using SIMCA 14.1 (Umetrics Inc., Sweden) (Hu et al., 2022). A hierarchical clustering heat map was generated using the TBtools (Chen et al., 2020a).

## 3 Results

### 3.1 Fruit morphological index analysis

The appearance characteristics of *Zhizi* and *Shuizhizi* mainly included obovate and round shapes, of which S1–S18 and S19–S25 represent obovate and round, respectively. These samples were categorized into seven length grades labeled A–G, where S1 falls into grade A (5.3 cm < L ≤ 6.3 cm), S2–S3 in grade B (4.3 cm < L ≤ 5.3 cm), S4–S5 in grade C (3.3 cm < L ≤ 4.3 cm), S6–S8 in grade D (2.7 cm < L ≤ 3.3 cm), S9–S14 in grade E (2.1 cm < L ≤ 2.7 cm), S15–S22 in grade F (1.5 cm < L ≤ 2.1 cm), and S23–S25 in grade G (0.8 cm < L ≤ 1.5 cm). These types are presented in **Supplementary Table 1** and **Figure 1**, where clear differences in the appearance traits of *Zhizi* and *Shuizhizi* fruits are evident.

For each fruit, the length, maximum diameter, ratio of length to maximum diameter, weight, volume, and density were considered as indices for analyzing the appearance characteristics. The data underwent analysis of ANOVA, and Duncan’s multiple range test results are presented in **Table 1**. The findings indicated that *Shuizhizi* was generally longer than *Zhizi*, although their length ranges overlapped. Significant differences were observed in the length of samples from different length grades. The ratio of fruit length to its maximum diameter served as an indicator of fruit appearance type, with a ratio closer to 1 suggesting a more rounded fruit. Significantly different ratios were observed between obovate and round *Zhizi* (p < 0.05). Round fruits exhibited a notably smaller ratio of length to maximum fruit diameter compared to obovate fruits. The experimental results supported the reliability of sample classification based on appearance. Additionally, for *Shuizhizi*, longer samples tended to have a larger ratio of length to maximum fruit diameter and heavier weight. When comparing *Shuizhizi* with *Zhizi* of the same length grade, *Shuizhizi* exhibited a larger volume and lower density than *Zhizi*.

**Table 1:** Morphological features of *Zhizi* and *Shuizhizi*.

No.	Species	Length grade	Length (cm)	Diameter (cm)	Length/diameter	Weight (g)	Volume (mL)	Density (g/mL)
S1	*Shuizhizi*	A (5.3 cm < L ≤ 6.3 cm)	5.808 ± 0.373^a^	2.196 ± 0.226^a^	2.658 ± 0.152^a^	3.77 ± 0.064^a^	7.56 ± 0.245^a^	0.50 ± 0.008^k^
S2		B (4.3 cm < L ≤ 5.3 cm)	4.756 ± 0.162^b^	1.981 ± 0.278^b^	2.449 ± 0.372^b^	3.15 ± 0.062^b^	5.88 ± 0.139^b^	0.54 ± 0.011^i-k^
S3		B (4.3 cm < L ≤ 5.3 cm)	4.559 ± 0.156^c^	1.924 ± 0.139^b^	2.380 ± 0.186^bc^	2.75 ± 0.035^c^	4.55 ± 0.233^c^	0.61 ± 0.029^d-j^
S4		C (3.3 cm < L ≤ 4.3 cm)	4.153 ± 0.233^d^	1.896 ± 0.178^b^	2.205 ± 0.199^d^	2.59 ± 0.043^d^	4.63 ± 0.132^c^	0.56 ± 0.070^h-k^
S5		C (3.3 cm < L ≤ 4.3 cm)	3.735 ± 0.130^e^	1.669 ± 0.186^c^	2.269 ± 0.313^cd^	2.52 ± 0.042^d^	3.94 ± 0.244^d^	0.64 ± 0.049^d-h^
S6		D (2.7 cm < L ≤ 3.3 cm)	3.100 ± 0.149^f^	1.627 ± 0.142^c^	1.922 ± 0.224^e^	1.50 ± 0.063^f^	2.66 ± 0.057^e^	0.56 ± 0.032^h-k^
S7	*Zhizi*	D (2.7 cm < L ≤ 3.3 cm)	2.954 ± 0.139^g^	1.513 ± 0.096^d^	1.956 ± 0.107^e^	1.60 ± 0.016^f^	2.19 ± 0.036^f^	0.73 ± 0.006^a-c^
S8		D (2.7 cm < L ≤ 3.3 cm)	2.889 ± 0.121^g^	1.527 ± 0.136^d^	1.906 ± 0.194^e^	1.73 ± 0.145^e^	2.33 ± 0.074^f^	0.74 ± 0.058^ab^
S9		E (2.1 cm < L ≤ 2.7 cm)	2.425 ± 0.111^h-j^	1.389 ± 0.121^e-g^	1.757 ± 0.160^fg^	1.17 ± 0.079^h^	1.70 ± 0.122^g^	0.69 ± 0.046^b-d^
S10		E (2.1 cm < L ≤ 2.7 cm)	2.493 ± 0.148^h^	1.438 ± 0.088^d-f^	1.736 ± 0.102^f-h^	1.30 ± 0.082^g^	1.71 ± 0.037^g^	0.76 ± 0.063^a^
S11		E (2.1 cm < L ≤ 2.7 cm)	2.446 ± 0.136^hi^	1.394 ± 0.094^e-g^	1.764 ± 0.182^fg^	0.93 ± 0.027^i^	1.43 ± 0.029^hi^	0.65 ± 0.025^c-f^
S12		E (2.1 cm < L ≤ 2.7 cm)	2.346 ± 0.165^i-k^	1.287 ± 0.103^g-i^	1.829 ± 0.157^ef^	0.78 ± 0.022^j^	1.31 ± 0.074^hi^	0.59 ± 0.043^f-j^
S13		E (2.1 cm < L ≤ 2.7 cm)	1.939 ± 0.128^l^	1.184 ± 0.138^i-k^	1.653 ± 0.182^g-i^	0.91 ± 0.016^i^	1.50 ± 0.058^gh^	0.61 ± 0.017^d-i^
S14		E (2.1 cm < L ≤ 2.7 cm)	2.291 ± 0.166^k^	1.337 ± 0.111^fg^	1.720 ± 0.149^f-h^	0.77 ± 0.020^j^	1.34 ± 0.071^hi^	0.57 ± 0.041^f-k^
S15		F (1.5 cm < L ≤ 2.1 cm)	1.947 ± 0.148^l^	1.222 ± 0.094^h-j^	1.599 ± 0.151^hi^	0.68 ± 0.057^j-k^	1.00 ± 0.031^kl^	0.68 ± 0.042^b-e^
S16		F (1.5 cm < L ≤ 2.1 cm)	1.856 ± 0.144^km^	1.192 ± 0.160^h-k^	1.573 ± 0.167^i^	0.49 ± 0.043^m^	0.83 ± 0.029^l-o^	0.59 ± 0.062^f-j^
S17		F (1.5 cm < L ≤ 2.1 cm)	2.310 ± 0.146^jk^	1.376 ± 0.090^e-g^	1.690 ± 0.186^f-i^	0.61 ± 0.017^l^	0.94 ± 0.054^k-m^	0.65 ± 0.045^d-g^
S18		F (1.5 cm < L ≤ 2.1 cm)	1.724 ± 0.131^no^	1.059 ± 0.082^l^	1.633 ± 0.126^g-i^	0.47 ± 0.070^m^	0.74 ± 0.132^m-o^	0.60 ± 0.014^e-j^
S19		F (1.5 cm < L ≤ 2.1 cm)	1.775 ± 0.146^m-o^	1.344 ± 0.097^fg^	1.325 ± 0.118^j^	0.65 ± 0.032^kl^	1.24 ± 0.076^ij^	0.53 ± 0.026^jk^
S20		F (1.5 cm < L ≤ 2.1 cm)	1.831 ± 0.155^l-n^	1.467 ± 0.113^de^	1.250 ± 0.085^jk^	0.73 ± 0.024^jk^	1.24 ± 0.048^ij^	0.59 ± 0.033^f-j^
S21		F (1.5 cm < L ≤ 2.1 cm)	1.692 ± 0.094^p^	1.295 ± 0.107^gh^	1.312 ± 0.090^j^	0.49 ± 0.009^m^	0.85 ± 0.040^k-n^	0.58 ± 0.031^f-k^
S22		F (1.5 cm < L ≤ 2.1 cm)	1.658 ± 0.100^p^	1.333 ± 0.078^fg^	1.248 ± 0.100^j^k	0.61 ± 0.021^l^	1.07 ± 0.005^jk^	0.57 ± 0.020^g-k^
S23		G (0.8 cm < L ≤ 1.5 cm)	1.344 ± 0.103^p^	1.091 ± 0.108^kl^	1.237 ± 0.092^jk^	0.37 ± 0.039^n^	0.68 ± 0.068^n-p^	0.54 ± 0.011^i-k^
S24		G (0.8 cm < L ≤ 1.5 cm)	1.289 ± 0.155°	1.036 ± 0.095^l^	1.244 ± 0.102^jk^	0.30 ± 0.023^n^	0.50 ± 0.027^p^	0.61 ± 0.055^d-j^
S25		G (0.8 cm < L ≤ 1.5 cm)	1.298 ± 0.107°	1.121 ± 0.105^j-l^	1.162 ± 0.091^k^	0.35 ± 0.016^n^	0.61 ± 0.042^op^	0.58 ± 0.020^f-j^

For each fruit morphological index, different letters in the upper right corner of the same columns denote significant differences (p < 0.05) among different samples. Using in the middle “-” omit the middle letter when there are more than two letters.

### 3.2 Analysis of the weight proportion of different parts of the fruit

Variations were observed in the weight proportion of pericarp, flesh, and seeds of *Gardenia* species fruit with different appearance characters and from different regions. Detailed information is presented in **Figure 1**. Notably, the pericarp weight constituted 25.7% to 33.15% of the total *Zhizi* fruit weight, while for *Shuizhizi*, it accounted for 32.78% to 36.32%. The pericarp weight proportion of *Shuizhizi* tended to be higher than that of *Zhizi*, while the total weight proportions of the flesh and seeds in *Shuizhizi* were generally lower than those in *Shuizhizi* compared to *Zhizi*. As the length grade of *Shuizhizi* decreased (**Figures 1A–D1**), there was a corresponding increase in the weight proportion of seeds and a decrease in the weight proportion of pericarps. The weight proportion of flesh changed with the proportions of pericarps and seeds. When the length ranges of *Zhizi* and *Shuizhizi* were the same (**Figures 1D1, 2**), the weight proportions of the flesh and seeds in *Zhizi* were higher than those in *Shuizhizi*, while the weight proportion of the pericarp was lower than that of *Shuizhizi*.

In various length grades of *Zhizi* from the Fujian production region (**Figures 1D2, E1**), shorter lengths correlated with higher weight proportion of seeds and lower weight proportions of flesh and pericarp. Across different length grades of obovate *Zhizi* from the Jiangxi production region (**Figures 1E2, F1**), decreasing lengths corresponded to lower weight proportions of seeds and higher weight proportions of flesh and pericarp. At the same length grade (**Figures 1E1, 2**), *Zhizi* sourced from Jiangxi showed a higher weight proportion of the pericarp and flesh compared to that from the Fujian. Conversely, the proportion of seeds weight was lower in Jiangxi-produced *Zhizi*. As the length of the round *Zhizi* decreased, there was a notable increase in the weight ratio of the pericarp; while the weight ratio of the flesh and seeds remained relatively low (**Figures 1F2, G**). Conversely, at identical length grades for obovate and round *Zhizi* (**Figures 1F1, 2**), obovate *Zhizi* exhibited relatively higher ratios of pericarp and flesh, alongside a relatively lower weight ratio of seeds.

### 3.3 Optimization of extraction solvents and UHPLC-MS/MS conditions

In UHPLC-MS/MS experiments, various extraction solvents, including 50% methanol, 70% methanol, and 70% ethanol, were employed for sample extraction. The results revealed that methanol yielded a superior extraction effect compared to ethanol. While the iridoids were more efficiently extracted with the 50% methanol solution, other components exhibited more comprehensive extraction in the 70% methanol solution. Ultrasonic extraction for either 30 or 40 minutes showed no significant difference. After careful consideration, 70% methanol was chosen for extraction, and ultrasonication for 30 minutes was deemed optimal. The optimized mass detection parameters for each analyte and representative chromatogram are presented in **Table 2** and **Supplementary Figure 2**, respectively.

**Table 2:** MS parameters of 13 investigated compounds.

No.	Analyte	Rt (min)	Formula	Q1 (m/z)	Q3 (m/z)	DP (V)	CE (eV)
1	Protocatechuic acid (CAS No. 99-50-3)	3.46	C_7_H_6_O_4_	152.9	109	-45.6	-20
2	Shanzhiside (CAS No. 29836-27-9)	3.61	C_16_H_24_O_11_	391	228.7	-101	-27
3	Geniposidic acid (CAS No. 27741-01-1)	3.7	C_16_H_22_O_10_	373.1	123	-76	-27
4	Deacetylasperulosidic acid methyl ester (CAS No. 52613-28-2)	4.2	C_17_H_24_O_11_	403.4	241	-42	-12
5	Neochlorogenic acid (CAS No. 906-33-2)	4.37	C_16_H_18_O_9_	353.1	190.8	-73	-27
6	Chlorogenic acid (CAS No. 327-97-9)	6.57	C_16_H_18_O_9_	353	191.1	-63	-32
7	Caffeic acid (CAS No. 331-39-5)	7.12	C_9_H_8_O_4_	178.9	134.9	-66	-22
8	Genipin 1-gentiobioside (CAS No. 29307-60-6)	7.88	C_23_H_34_O_15_	549	224.8	-65	-20
9	Geniposide (CAS No. 24512-63-8)	8.82	C_17_H_24_O_10_	387.3	224.9	-54	-12
10	Genipin (CAS No. 6902-77-8)	10.29	C_11_H_14_O_5_	225.1	123.1	-56	-10.5
11	Isoquercitrin (CAS No. 482-35-9)	11.81	C_21_H_20_O_12_	463.2	254.9	-134	-54.4
12	Crocin I (CAS No. 42553-65-1)	16.23	C_44_H_64_O_24_	975.4	651.2	-115	-28
13	Crocin II (CAS No. 55750-84-0)	17.84	C_38_H_54_O_19_	813.4	489	-127	-25

### 3.4 Method validation

In this study, the method’s performance was thoroughly validated, encompassing linearity, LOD, LOQ, precision, stability, and recovery. The results are summarized in **Table 3**. All the standard curves demonstrated excellent linear relationships (r > 0.999) across a relatively broad concentration ranges. The LOD and LOQ for the 13 compounds ranged from 0.0003 to 0.5 µg/mL and 0.0005 to 1.2 µg/mL, respectively. Intra-day precision exhibited RSD values ranging between 0.92% and 4.57%, while inter-day precision ranged from 2.27% to 5.82%. The stability RSD values for the 13 compounds were all below 4.98%, and repeatability was less than 6.03%. Overall recoveries fell within the range of 95.23% to 104.51%, with RSD values below 4.8%. These results affirmed the suitability of the method for the simultaneous quantitative analysis of the 13 compounds in both *Zhizi* and *Shuizhizi*.

**Table 3:** Method validation of 13 compounds.

No.	Analyte	Calibration curves	r	Linear range (μg/mL)	LOD(μg/mL)	LOQ(μg/mL)	Precision(RSD % n=6)		Repeatability(RSD % n=6)	Stability (RSD % n=6)	Recovery(RSD % n=6)	
							intra-day	inter-day			Mean	RSD
1	Protocatechuic acid	y = 5000000x + 33376	0.9999	0.01-10	0.002	0.006	0.92	2.83	1.46	2.37	104.51	3.99
2	Shanzhiside	y = 152367x + 31867	0.9995	0.2-20	0.0048	0.011	1.13	2.3	1.97	2.77	103.37	2.12
3	Geniposidic acid	y = 747106x + 3805.7	0.9999	0.05-10	0.008	0.018	1.05	2.76	1.48	2.94	102	3.54
4	Deacetylasperulosidic acid methyl ester	y = 2397x + 322.54	0.9999	0.5-40	0.05	0.15	3.51	4.49	4.05	4.08	101.32	3.81
5	Neochlorogenic acid	y = 220410x - 35120	0.9996	0.2-40	0.01	0.032	1.82	2.68	1.04	2.82	100.53	3.37
6	Chlorogenic acid	y = 1000000x + 674965	0.9995	0.1-100	0.001	0.003	1.53	2.27	2.3	1.72	103.13	1.93
7	Caffeic acid	y = 9000000x - 265231	0.9993	0.1-2	0.025	0.075	1.61	2.75	3.03	3.78	95.23	4.07
8	Genipin 1-gentiobioside	y = 35625x + 33324	0.9998	0.1-200	0.0067	0.02	3.46	4.68	4.31	4.98	101.8	4.17
9	Geniposide	y = 3994.7x + 310283	0.9996	100-600	0.5	1.2	1.53	2.98	1.32	3.32	97.16	4.27
10	Genipin	y= 3000000x + 5832.7	1	0.001-1	0.0003	0.0005	1.48	3.08	1.13	2.62	98.25	1.16
11	Isoquercitrin	y = 239197x - 3581.1	1	0.02-10	0.001	0.004	4.57	5.76	5.8	2.76	100.47	3.31
12	Crocin I	y = 13469x + 52811	0.9998	1-250	0.003	0.02	1.45	3.15	0.86	1.53	102.65	4.56
13	Crocin II	y = 6206.3x - 2642	0.9996	0.5-20	0.075	0.1125	4.3	5.82	6.03	2.7	96.11	4.8

### 3.5 Comparative content analysis of 13 compounds in *Zhizi* and *Shuizhizi*

**Figure 2:**
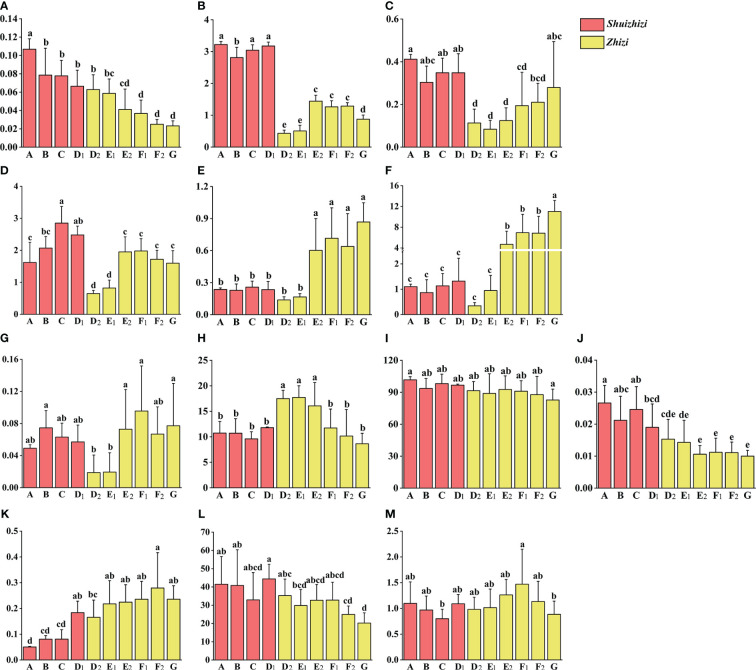
Chemical component content in different length grades of *Zhizi* and *Shuizhizi* (mg/g). The different letters within each graph mean statistically significant differences (p < 0.05) by Duncan’s multiple range test among samples about different length grades of *Zhizi* and *Shuizhizi*. The content data is marked with letters a, b, c, etc. from high to low. **(A)** Protocatechuic acid; **(B)** Shanzhiside; **(C)** Geniposidic acid; **(D)** Deacetylasperulosidic acid methyl ester; **(E)** Neochlorogenic acid; **(F)** Chlorogenic acid; **(G)** Caffeic acid; **(H)** Genipin 1-gentiobioside; **(I)** Geniposide; **(J)** Genipin; **(K)** Isoquercitrin; **(L)** Crocin I; **(M)** Crocin II.

To further conduct quantitative analysis and investigate potential differences in compound contents between *Zhizi* and *Shuizhizi* with distinct appearance traits, we employed the UHPLC-QQQ-MS/MS method. This allowed us to simultaneously determine the levels of 13 chemical components in both *Zhizi* and *Shuizhizi*. The targeted components included six iridoids (geniposide, genipin, shanzhiside, geniposidic acid, genipin 1-gentiobioside, and deacetylasperulosidic acid methyl ester), two pigments (crocin I and crocin II), four organic acids (caffeic acid, chlorogenic acid, neochlorogenic acid and protocatechuic acid) and one flavonoid (isoquercitrin). The results depicting the content of compounds in different types of *Zhizi* and *Shuizhizi* are illustrated in **Figure 2**. The detailed content values for the 13 compounds in the 25 batches of *Zhizi* and *Shuizhizi* samples can be found in **Supplementary Table 2**.

#### 3.5.1 Comparative analysis of 13 components in different types of *Zhizi* and *Shuizhizi*

Iridoids play a crucial role as chemical constituents of Gardeniae Fructus. Notably, there was no significant difference in geniposide content between *Zhizi* and *Shuizhizi*. However, *Shuizhizi* exhibited a higher content of iridoids, such as geniposidic acid, genipin, shanzhiside, and deacetylasperulosidic acid methyl ester, compared to *Zhizi*. As the fruit length of the *Gardenia* species decreased, the protocatechuic acid content decreased. Additionally, a decline in length corresponded to a decrease in the chlorogenic acid content in *Zhizi*.

In *Shuizhizi*, the contents of geniposide, geniposidic acid, genipin, and protocatechuic acid in grade A were slightly higher than in other grades. However, the content of deacetylasperulosidic acid methyl ester in grade C surpassed that in grades A and B, and the content of isoquercitrin in grade D was higher than in grades A–C. In addition, the content of crocin I and crocin II in grade C was slightly lower than in grades A, B, and D. When *Zhizi* and *Shuizhizi* were in length grade D, the content of genipin 1-gentiobioside in *Zhizi* surpassed that in *Shuizhizi*. Conversely, the contents of geniposidic acid, shanzhiside, and deacetylasperulosidic acid methyl ester in *Shuizhizi* were mostly higher than in *Zhizi*.

Comparing obovate and round *Zhizi* with the same length grade, the contents of caffeic acid, crocin I, and crocin II were slightly higher in obovate *Zhizi*. However, isoquercitrin showed slightly lower content in obovate *Zhizi*. With decreasing length grades in round *Zhizi*, the geniposide, shanzhiside, crocin I, and crocin II contents exhibited a decreasing trend, while chlorogenic acid and caffeic acid contents showed increasing trends. *Zhizi* from Fujian demonstrated a higher content of genipin 1-gentiobioside and protocatechuic acid, but lower contents of geniposidic acid, shanzhiside, deacetylasperulosidic acid methyl ester, caffeic acid, chlorogenic acid, and neochlorogenic acid compared to most *Zhizi* from Jiangxi. In the E length grade, *Zhizi* from Fujian exhibited lower contents of shanzhiside, deacetylasperulosidic acid methyl ester, chlorogenic acid, and neochlorogenic acid than *Zhizi* from Jiangxi. However, the contents of genipin 1-gentiobioside and protocatechuic acid were slightly higher in *Zhizi* from Fujian.

The quantitative results indicated that most iridoids in *Shuizhizi* were higher than those in *Zhizi*. Furthermore, there are differences in the chemical component content between *Zhizi* from Jiangxi and Fujian. Additionally, a decreasing trend in the protocatechuic acid was noted with decreasing length in both *Zhizi* and *Shuizhizi*.

#### 3.5.2 Multivariate statistical analysis

**Figure 3:**
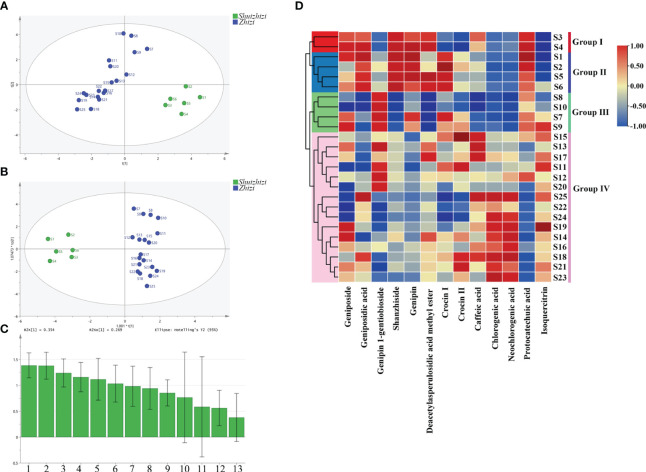
Multivariate statistical analysis and heatmap cluster analysis **(A)** PCA score plot; **(B)** OPLS-DA score plot; **(C)** the VIP plot; and **(D)** Hierarchical clustering analysis heat maps. The numbers in the VIP plot represent 13 compounds, (1) Shanzhiside; (2) Genipin; (3) Protocatechuic acid; (4) Geniposidic acid; (5) Isoquercitrin; (6) Chlorogenic acid; (7) Neochlorogenic acid; (8) Deacetylasperulosidic acid methyl ester; (9) Genipin 1-gentiobioside; (10) Crocin I; (11) Geniposide; (12) Caffeic acid; (13) Crocin II.

PCA and OPLS-DA, as commonly used multivariate statistical analysis methods, can achieve sample classification. The content of 13 compounds in 25 batches of *Zhizi* and *Shuizhizi* was used for PCA analysis, and the results showed that 19 batches of *Zhizi* and 6 batches of *Shuizhizi* could be well distinguished (**Figure 3A**), consistent with prior studies (Cao et al., 2021a). In order to further understand which compounds have a significant contribution in distinguishing between *Zhizi* and *Shuizhizi*, those compounds datas were used for OPLS-DA analysis (**Figure 3B**). In the established statistical model, the R^2^Y, and Q^2^ were 0.95 and 0.931, respectively. At the same time, 200 permutation tests were carried out to verify the OPLS-DA model. The vertical intercept values of R^2^ and Q^2^ were 0.18 and -0.644, respectively. The above data indicated that the established OPLS-DA model had good quality. Through OPLS-DA analysis, each compound can obtain a variable importance of projection (VIP) value. The larger the VIP value, the greater the contribution of the substance to distinguishing different groups. When the VIP value of a compound exceeded 1, it was considered to carry the most relevant information for group classification. The OPLS-DA score plot (**Figure 3B**) showed that *Zhizi* and *Shuizhizi* were completely separated, and the VIP value (**Figure 3C**) indicated that three iridoids (geniposidic acid, shanzhiside, genipin), two organic acids (chlorogenic acid, protocatechuic acid), and one flavonoids (isoquercetin) had a greater contribution to distinguishing them.

The hierarchical clustering analysis heatmap effectively classifies different samples based on the similarity of their chemical components. In this study, a hierarchical cluster analysis heatmap was generated for the 13 chemical components across 25 batches of *Zhizi* and *Shuizhizi* samples. The results are presented in **Figure 3D**. The colors in the figure indicate the content of each compound, ranging from low (blue) to high (red). The samples were distinctly grouped into four categories. Group I (S3 and S4) and Group II (S1, S2, S5, and S6) comprised *Shuizhizi* samples from Jiangxi, Group III (S7–S10) consisted of *Zhizi* samples from Fujian, and Group IV (S11–S25) included *Zhizi* samples from Jiangxi. In this study, all *Zhizi* (Groups III and IV) and *Shuizhizi* (Groups I and II) samples could be distinguished based on their chemical composition, consistent with the results of PCA and OPLS-DA. Moreover, *Zhizi* from Jiangxi (Group IV) and Fujian (Group III) were successfully differentiated. *Shuizhizi* was further divided into two groups, and compared with Group I, Group II exhibited higher levels of genipin 1-gentiobioside, crocin I, and crocin II, along with lower levels of caffeic acid. This distinction might be attributed to the fact that there are two main cultivated varieties of *Shuizhizi*, whose fruits are often mixed together for use.

#### 3.5.3 Analysis of different parts of *Zhizi* and *Shuizhizi*

To delve deeper into the variations in compound content across different parts of *Zhizi* and *Shuizhizi*, the UHPLC-QQQ-MS/MS method was employed to simultaneously determine the content of 13 components in distinct parts (pericarp, flesh, and seeds) of both *Zhizi* and *Shuizhizi*.

The total content of various compounds in the pericarp, flesh, and seeds of *Zhizi* and *Shuizhizi* is depicted in **Supplementary Figure 3**. Notably, the total contents of six iridoids and two pigments were higher in the flesh and seeds, while lower in the pericarp of both *Zhizi* and *Shuizhizi*. Conversely, the total contents of four organic acids and one flavonoid were highest in the pericarp, with lower contents in the flesh and seeds. Specifically, the total content of six iridoids in the seeds of *Shuizhizi* surpassed that in the *Zhizi*. Moreover, the total content of the two pigments in the flesh of *Zhizi* was significantly higher than in *Shuizhizi*. Additionally, the total contents of the four organic acids and one flavonoid in the pericarp, flesh, and seeds of *Zhizi* were higher than those in the corresponding parts of *Shuizhizi*.

**Figure 4:**
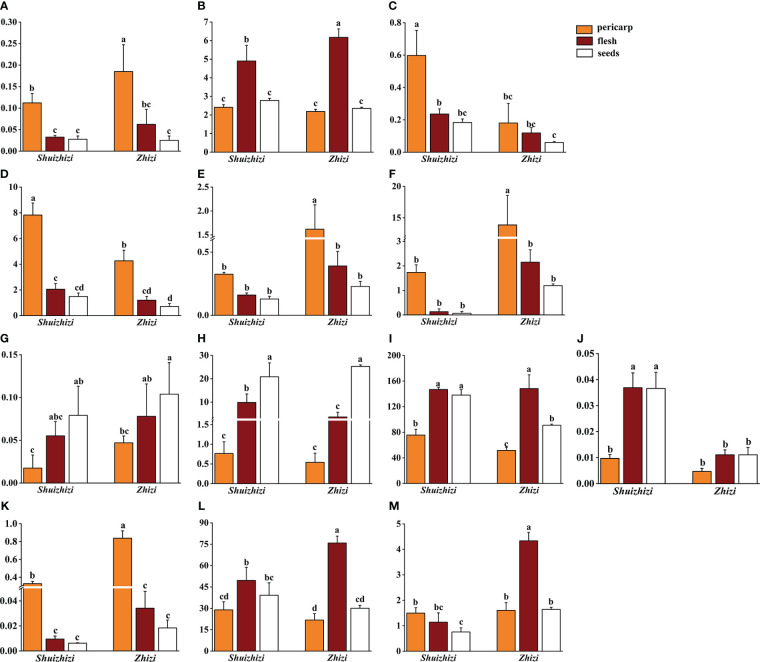
Compound content in different parts (pericarp, flesh, and seeds) of *Zhizi* and *Shuizhizi.* The different letters within each graph denote significant differences (p < 0.05) by Duncan’s multiple range test among samples about different parts of *Zhizi* and *Shuizhizi.* The content data is marked with letters a, b, c, etc. from high to low. **(A)** Protocatechuic acid; **(B)** Shanzhiside; **(C)** Geniposidic acid; **(D)** Deacetylasperulosidic acid methyl ester; **(E)** Neochlorogenic acid; **(F)** Chlorogenic acid; **(G)** Caffeic acid; **(H)** Genipin 1-gentiobioside; **(I)** Geniposide; **(J)** Genipin; **(K)** Isoquercitrin; **(L)** Crocin I; **(M)** Crocin II.

The distributions of the 13 compounds in the pericarp, flesh, and seeds of *Zhizi* and *Shuizhizi* exhibited a similar pattern, as depicted in **Figure 4**. For iridoids and pigments, geniposide, genipin 1-gentiobioside, shanzhiside, genipin, and crocin I showed higher contents in the flesh and seeds and lower in the pericarp. Conversely, the contents of geniposidic acid and deacetylasperulosidic acid methyl ester were higher in the pericarp and lower in the flesh and seeds in both *Zhizi* and *Shuizhizi*. Genipin 1-gentiobioside demonstrated the highest content in the seeds, followed by the flesh, and the lowest in the pericarp for both *Zhizi* and *Shuihizi*. Crocin II exhibited higher content in the pericarp and flesh of *Shuizhizi*, and the lowest in the seeds. In *Zhizi*, crocin II content in the flesh was higher than in the pericarp and seeds. The contents of organic acids (chlorogenic acid, neochlorogenic acid, and protocatechuic acid) and one flavonoid (isoquercitrin) were highest in the pericarps of both *Zhizi* and *Shuizhizi*, with lower contents in the flesh and seeds. Conversely, caffeic acid content exhibited the following order in *Zhizi* and *Shuizhizi*: seeds > flesh > pericarp. Furthermore, the chlorogenic acid, neochlorogenic acid, protocatechuic acid, and isoquercitrin contents in the pericarp of *Zhizi* were significantly higher than those in *Shuizhizi*.

## 4 Discussion

### 4.1 Comprehensive analysis of various *Zhizi* and *Shuizhizi* types: foundations for *Shuizhizi* development and utilization

Gardeniae Fructus, known as *Zhizi*, boasts a medicinal and dyeing history of thousands of years in China. Ancient herbalists recognized two distinct appearances of fruits of *Gardenia* species — a larger one primarily used for dyeing and a rounder, smaller variant for medicinal purposes. Although most of the fruit morphologies of *Zhizi* and *Shuizhizi* are similar, *Shuizhizi* is generally larger and longer than *Zhizi*, with occasional size overlap between the two (Chen, 2018), as seen in samples S6, S7, and S8 in this study. Currently, a variety of appearance parameters have been used to differentiate medicinal fruits from different varieties or locations (Huang et al., 2022; Ma et al., 2022). Therefore, beyond solely measuring the length and maximum diameter of the fruits, this study incorporated measurements such as weight, volume, and density to comprehensively analyze the appearance traits of *Zhizi* and *Shuizhizi*. This approach provides a more intuitive understanding of their respective visual characteristics.

When the lengths of *Zhizi* and *Shuizhizi* were similar, *Zhizi* exhibited smaller volume and higher density. In addition to these differences in appearance, notable distinctions existed in the chemical composition between *Zhizi* and *Shuizhizi*. Some researchers have highlighted that the primary differentiators in *Zhizi* and *Shuizhizi* are mainly iridoids (Zhang et al., 2024). Ye et al. employed both non-targeted and targeted data analyses to accurately discern the differences in phytochemicals accumulated in the fruits of three *Gardenia* species, *Shuizhizi* and two kinds of *Zhizi* cultivars (Ye et al., 2022b). The 13 compounds quantified in this study, including six iridoids, four organic acids, two pigments, and one flavonoid effectively differentiated between the medicinal materials of *Zhizi* and *Shuizhizi*, as well as between *Zhizi* sourced from Jiangxi and Fujian (**Figure 3**). A previous study showed that Gardeniae Fructus with leaner and longer fruit shapes exhibited a lower total content of six phenolic acids and rutin (Ye et al., 2022a). In our study, the protocatechuic acid content decreased with the decrease of length grade for both *Zhizi* and *Shuizhizi*. The chlorogenic acid content in *Zhizi* displayed an upward trend with decreasing length. *Zhizi* exhibits superior quality characterized by small, round, thin pericarps and red color (Liu and Peng, 2016; Rao et al., 2023). The results of our study indicated the inaccuracy of dividing Gardeniae Fructus grades solely based on length grade. This study provides a reference for commodity specification and grade division of *Zhizi*, and further investigates the classification of *Zhizi* and *Shuizhizi*.

Historical herbal texts mention the medical use of *Zhizi* while excluding *Shuizhizi* from medicinal applications. However, ancient prescriptions such as *Treatise on Febrile Diseases* include references to “*Fei Zhizi*” and “*Da Zhizi*” as medicine ingredients, indicating that *Shuizhizi* was indeed used in ancient medicine (Xu et al., 2020b). Iridoids stand as the primary active components in Gardeniae Fructus, with geniposide, an iridoid, serving as a quality evaluation marker in the Chinese Pharmacopoeia, which stipulates that the content of geniposide in Gardeniae Fructus should not be less than 1.8% (Committee for the Pharmacopoeia of PR China, 2020). In this study, both *Zhizi* and *Shuizhizi* met the geniposide content standard outlined in the Chinese Pharmacopoeia, and no significant difference was observed in the geniposide contents of *Zhizi* and *Shuizhizi*. In addition, *Shuizhizi* exhibits a range of other iridoids and pigments. The quantities of six iridoids in *Shuizhizi* were generally higher than those found in *Zhizi*, with the exception of geniposide and genipin 1-gentiobioside. Modern pharmacological studies have revealed significant diuretic and antihypertensive effects in the total extract of *Shuizhizi* and its ethyl acetate extracts (Fu et al., 2020b). *Shuizhizi* also demonstrates anti-allergic pharmacological activity, with geniposide being one of its main efficacious substances (Wang et al., 2020). Furthermore, certain terpenoids present in *Shuizhizi* exhibit renoprotective activity (Cao et al., 2021b). These findings provide a foundation for the judicious use of *Shuizhizi*, apart from its use as a dye, and highlight its potential medical applications. Yet, further research is needed to fully explore its medicinal value.

### 4.2 Implications of chemical component distribution variation in different parts of *Gardenia* fruit for clinical drug usage

Secondary metabolites in medicinal plants are unevenly distributed across different organs and tissues (Chen et al., 2019; Zhao et al., 2021). In terms of plant fruits, variations in chemical components exist across different parts of the fruit, as observed in *Juglans regia* L. and *Citrus reticulata* Blanco (Bourais et al., 2022; Yang et al., 2022). Previous studies have found that representative iridoids (geniposidic acid, genipin 1-gentiobioside, and geniposide) and pigments (crocin I and crocin II) were predominantly concentrated in the kernel of *Zhizi*, while organic acids and flavonoids (chlorogenic acid and rutin) were mainly distributed in the pericarp of *Zhizi* (Wu et al., 2014). In this study, the fruits of *Zhizi* and *Shuizhizi* were divided into three parts: pericarp, flesh, and seeds. It was observed that the weight ratios of pericarp, flesh, and seeds varied among different character types of *Zhizi* and *Shuizhizi* (**Figure 1**). The weight ratio of *Zhizi* pericarps was mostly smaller than that of *Shuizhizi* pericarps, consistent with the notion of favoring thin pericarps for medicinal applications (Rao et al., 2023). Iridoids constitute the key chemical components of *Zhizi*, exhibiting the highest content and significant biological activity (Chen et al., 2020b). In this study, geniposidic acid and deacetylasperulosidic acid methyl ester were found to be primarily distributed in the pericarp, while geniposide, genipin, genipin 1-gentiobioside and shanzhiside were predominantly concentrated in the flesh and seeds. The two pigments were mostly concentrated in the flesh and seeds of *Zhizi* and *Shuizhizi*, whereas the four organic acids and one flavonoid mainly accumulated in the pericarps of *Zhizi* and *Shuizhizi*.

Previous studies shown that, the distribution differences of compounds in *Zhizi* might be closely related to the tissue-specific expression of genes involved in the synthesis or regulation of these compounds. Xu et al. found significant differences in the accumulation patterns of geniposide and crocin during fruit ripening in *Zhizi* (Xu et al., 2023). Moreover, the enrichment degree of iridoids such as geniposide in the kernel was significantly higher than that in the peel at the growth and maturation stage, which was highly consistent with the expression level of predicted genes of main enzyme G8O (8-hydroxygeraniol dehydrogenase) in its metabolic pathway. The high expression of *G8Os* in the kernel of *Zhizi* might be the reason for the high content of geniposide in the kernel (Pan et al., 2021). Recently, there have been many studies on the metabolic pathway of crocin biosynthesis in *Zhizi* (Xu et al., 2020a; Shen et al., 2022). During the development of gardenia fruit, when the fruit color turns red, key enzyme genes involved in the biosynthesis of crocin exhibit significantly high expression levels (Zhang et al., 2023). The accumulation of crocin has guiding significance for determining the appropriate harvesting time of fruits.

Iridoids and pigments constitute the key chemical components of *Zhizi*, exhibiting the highest content and significant biological activity (Chen et al., 2020b; Wang et al., 2024). In addition, the main components of the two categories exhibited lower distribution in its pericarp, which aligns with the traditional wisdom that emphasizes the superiority of *Zhizi* with a lighter pericarp. In addition to using the whole fruit as a medicine, the historical use of Gardeniae Fructus’s pericarp and kernel (Li et al., 2020) is supported by traditional texts like *Lei Gong Pao Zhi Lun*, which recommends peeling and whisking *Zhizi* first and using the kernel for medicinal purposes (Lei, 1985). The ancient *Compendium of Materia Medica* documents *Zhizi*’s dual role in shelling or removing shells to treat different diseases (Li, 1996). Recent research reveals that the pericarp of Gardeniae Fructus is a potent source of dietary fibers and exhibits hypoglycemic properties (Meng et al., 2021). Findings from this study indicated that *Zhizi*’s flesh and seeds contain higher concentrations of iridoids and pigments, while organic acids and flavonoids are predominantly present in its pericarp. These insights offer a valuable reference for exploring the distinctive components across different parts of *Zhizi*. Meanwhile, further genetic evidence is necessary to elucidate the underlying mechanisms behind the medicinal differences in its different parts.

## 5 Conclusion

This study investigated obovate and round *Zhizi* and *Shuizhizi* fruits by categorizing them into seven different grades (A–G) based on their length. Beyond exploring appearance traits and weight distributions of the pericarp, flesh, and seeds in various types of *Zhizi* and *Shuizhizi*, an effective UHPLC-QQQ-MS/MS method was developed to simultaneously quantify 13 main chemical components in the entire fruit and its different parts. The findings revealed that, generally, *Shuizhizi* was larger and longer than *Zhizi*, albeit with occasional crossovers in fruit size ranges. The weight proportion of the *Shuizhizi* pericarp was mostly higher than that of the *Zhizi* pericarp. Quantitative analysis of the 13 main chemical components indicated significant differences between *Zhizi* and *Shuizhizi*. PCA, OPLS-DA, and heatmap cluster analysis distinctly divided *Shuizhizi* and *Zhizi*, and *Zhizi* from different regions also showed clear distinctions through heatmap cluster analysis. Except for geniposide and genipin 1-gentiobioside, the contents of other four iridoids in *Shuizhizi* were generally higher than those in *Zhizi*. Furthermore, the distribution of the 13 chemical components in different parts of *Zhizi* and *Shuizhizi* remained consistent. Most iridoids and pigments were concentrated in the seeds and flesh, while organic acids and a flavonoid were primarily found in the pericarps for both. This study provides insights for classifying commodity specifications and grades of Gardeniae Fructus. Additionally, it offers guidance for the clinical application of *Shuizhizi* and the scientifically rational utilization of different parts of *Zhizi*.

## Data availability statement

The original contributions presented in the study are included in the article/**Supplementary Material**. Further inquiries can be directed to the corresponding authors.

## Author contributions

HQ: Data curation, Methodology, Writing – original draft. YH: Data curation, Methodology, Writing – original draft. ZW: Resources, Writing – original draft. AR: Resources, Writing – original draft. HZ: Writing – original draft. SC: Data curation, Methodology, Supervision, Writing – original draft, Writing – review & editing. HP: Supervision, Writing – review & editing, Writing – original draft.
