# Supplementary material for: Comprehensive quality evaluation of different types of Gardeniae Fructus (Zhizi) and Shuizhizi based on LC-MS/MS
Source: Front Plant Sci. 2024 Feb 27;15:1346591. doi: 10.3389/fpls.2024.1346591 (PMC10927785; doi:10.3389/fpls.2024.1346591)
Supplement: Supplementary file 1 [file DataSheet_1.zip › Supplementary Figures.doc]

***Supplementary Material***

# Supplementary Figures

**Supplementary Figure 1.** Structures of the 13 reference compounds. **(A)** Shanzhiside; **(B)** Geniposide; **(C)** Geniposidic acid; **(D)** Isoquercitrin; **(E)** Chlorogenic acid; **(F)** Neochlorogenic acid; **(G)** Deacetylasperulosidic acid methyl ester; **(H)** Caffeic acid; **(I)** Protocatechuic acid; **(J)** Genipin 1-gentiobioside; **(K)** Genipin; **(L)** Crocin I; **(M)** Crocin II.

**Supplementary Figure 2.** Typical chromatograms of 13 reference compounds.

**Supplementary Figure 3.** Total contents of 4 kinds of compounds in different parts of *Zhizi* and *Shuizhizi*. **(A)** Total content of 6 kinds of total iridoids; **(B)** Total content of two pigments; **(C)** Total content of four organic acids; **(D)** One flavonoid content.

**Supplementary Figure 1.** Structures of the 13 reference compounds. **(A)** Shanzhiside; **(B)** Geniposide; **(C)** Geniposidic acid; **(D)** Isoquercitrin; **(E)** Chlorogenic acid; **(F)** Neochlorogenic acid; **(G)** Deacetylasperulosidic acid methyl ester; **(H)** Caffeic acid; **(I)** Protocatechuic acid; **(J)** Genipin 1-gentiobioside; **(K)** Genipin; **(L)** Crocin I; **(M)** Crocin II.

**Supplementary Figure 2.** Typical chromatograms of 13 reference compounds

**Supplementary Figure 3.** Total contents of 4 kinds of compounds in different parts of *Zhizi* and *Shuizhizi.* (**A)** Total content of 6 kinds of total iridoids; **(B)** Total content of two pigments; **(C)** Total content of four organic acids; **(D)** One flavonoid content.
